# Individual 5-Year Lung Cancer Risk Prediction Model in Korea Using a Nationwide Representative Database

**DOI:** 10.3390/cancers13143496

**Published:** 2021-07-13

**Authors:** Yohwan Yeo, Dong Wook Shin, Kyungdo Han, Sang Hyun Park, Keun-Hye Jeon, Jungkwon Lee, Junghyun Kim, Aesun Shin

**Affiliations:** 1 Department of Family Medicine & Supportive Care Center, Samsung Medical Center, Sungkyunkwan University School of Medicine, Seoul 06351, Korea; yohwan.yeo@samsung.com; 2 Department of Preventive Medicine, Seoul National University College of Medicine, Seoul 03080, Korea; shinaesun@snu.ac.kr; 3 Department of Clinical Research Design & Evaluation, Samsung Advanced Institute for Health Science & Technology (SAIHST), Sungkyunkwan University, Seoul 06351, Korea; 4 Department of Digital Health, Samsung Advanced Institute for Health Science & Technology (SAIHST), Sungkyunkwan University, Seoul 06351, Korea; 5 Department of Statistics and Actuarial Science, Soongsil University, Seoul 06978, Korea; 6 Department of Medical Statistics, College of Medicine, Catholic University of Korea, Seoul 06591, Korea; ujk8774@catholic.ac.kr; 7 Department of Family Medicine, CHA Gumi Medical Center, Gumi 39295, Korea; kh1228@chamc.co.kr; 8 Bucheon Geriatric Medical Center, Bucheon 14478, Korea; jklee@skku.edu; 9 Department of Family Medicine, Samsung Medical Center, Sungkyunkwan University School of Medicine, Seoul 06351, Korea; 10 Division of Pulmonary and Critical Care Medicine, Department of Internal Medicine, National Medical Center, Seoul 04564, Korea; junghyun.kim@nmc.or.kr

**Keywords:** lung cancer, prediction, personalized risk, decision aids

## Abstract

**Simple Summary:**

From the representative data in Korea, we developed individual lung cancer risk prediction model of Korean adults. Our model would serve as a tool to screen high-risk individuals who would benefit from participating in lung cancer screening in a clinical setting applicable to health examinees or the general adult population. We believe that interactive approaches between healthcare providers and examinees using an easily accessible and visualized risk score can be used for the development of health policies for lung cancer prevention.

**Abstract:**

Early detection of lung cancer by screening has contributed to reduce lung cancer mortality. Identifying high risk subjects for lung cancer is necessary to maximize the benefits and minimize the harms followed by lung cancer screening. In the present study, individual lung cancer risk in Korea was presented using a risk prediction model. Participants who completed health examinations in 2009 based on the Korean National Health Insurance (KNHI) database (DB) were eligible for the present study. Risk scores were assigned based on the adjusted hazard ratio (HR), and the standardized points for each risk factor were calculated to be proportional to the b coefficients. Model discrimination was assessed using the concordance statistic (c-statistic), and calibration ability assessed by plotting the mean predicted probability against the mean observed probability of lung cancer. Among candidate predictors, age, sex, smoking intensity, body mass index (BMI), presence of chronic obstructive pulmonary disease (COPD), pulmonary tuberculosis (TB), and type 2 diabetes mellitus (DM) were finally included. Our risk prediction model showed good discrimination (c-statistic, 0.810; 95% CI: 0.801–0.819). The relationship between model-predicted and actual lung cancer development correlated well in the calibration plot. When using easily accessible and modifiable risk factors, this model can help individuals make decisions regarding lung cancer screening or lifestyle modification, including smoking cessation.

## 1. Introduction

Lung cancer is the leading cause of cancer death worldwide [1,2]. In Korea, there were 25,780 cases of lung cancer and 17,963 deaths from lung cancer in 2016 [2]. Although the lung cancer survival rate remains poor, a decrease in lung cancer incidence and mortality has been observed in Korea over the last few decades [3,4]. Early detection using low-dose chest computed tomography (CT) screening has contributed to the reduction in lung cancer mortality, as well as the introduction of new chemotherapy and molecular targeted agents. In 2019, the Korean national screening program for lung cancer was initiated for individuals >55 years of age and who currently smoke or have a smoking history (e.g., ≥30 pack-years of smoking and <15 years since quitting). However, identifying the appropriate population recommended for screening to maximize the efficacy of the screening program remains controversial.

The US National Lung Screening Trial (NLST) projected a 20% risk reduction in lung cancer mortality in high-risk patients when screened [5]. However, even within this high-risk population, 20% of participants at lowest risk of lung cancer in the NLST accounted for only 1% of the lung cancer deaths prevented when using CT screening [6], emphasizing the importance of having a precisely defined population for screening. Limiting screening to subjects at sufficiently high risk, who are most likely to benefit from screening, will maximize the benefit in terms of early detection and minimize the harm associated with detecting false positives among subjects at lower risk [7].

However, previous prediction models are predominantly from Western countries [8,9,10,11,12,13,14,15,16]. A previous prediction model in Korea showed good performance with a c-statistic of 0.871 and that early exposure to smoking is an important factor for developing lung cancer [17]. Unfortunately, the model was developed only for men due to insufficient data for smoking in women. Regarding the ethnic difference in lung cancer epidemiology in Korea compared with Western countries [18], development of an individualized risk prediction model for lung cancer and methods to identify high-risk groups that can be applied to the entire Korean population is necessary. Therefore, we developed a risk prediction model for lung cancer using representative data from a large population-based cohort in Korea.

## 2. Methods

### 2.1. Database Source

In the present retrospective cohort study, the Korean National Health Insurance (KNHI) database (DB) was used, which includes data on inpatient visits, outpatient visits, procedures, and prescription medications covered by the KNHI, a mandatory universal public health insurance system that covers the entire Korean population except for Medicaid beneficiaries in the lowest-income bracket (approximately 3% of the population). All Korean citizens are encouraged to receive regular biannual or pre-employment health evaluations provided by the KNHI. The KNHI DB contains a qualification DB (e.g., age, sex, income, region, and type of eligibility), a claims DB (e.g., general information on specifications; consultation statements; diagnosis statements defined by the International Classification of Diseases, 10th revision (ICD-10); and prescription statements), a health checkup DB, and death information. Medical history and alcohol, smoking, and exercise habits are collected using standardized self-reporting questionnaires. The KNHI DB has been widely used in various epidemiological and health policy studies [19,20]. Details of the DB profile are described elsewhere [21,22]. This study was approved by the institutional review board (IRB) of Samsung Medical Center (IRB file no. SMC 2017-12-039).

### 2.2. Study Population

**Figure 1:**
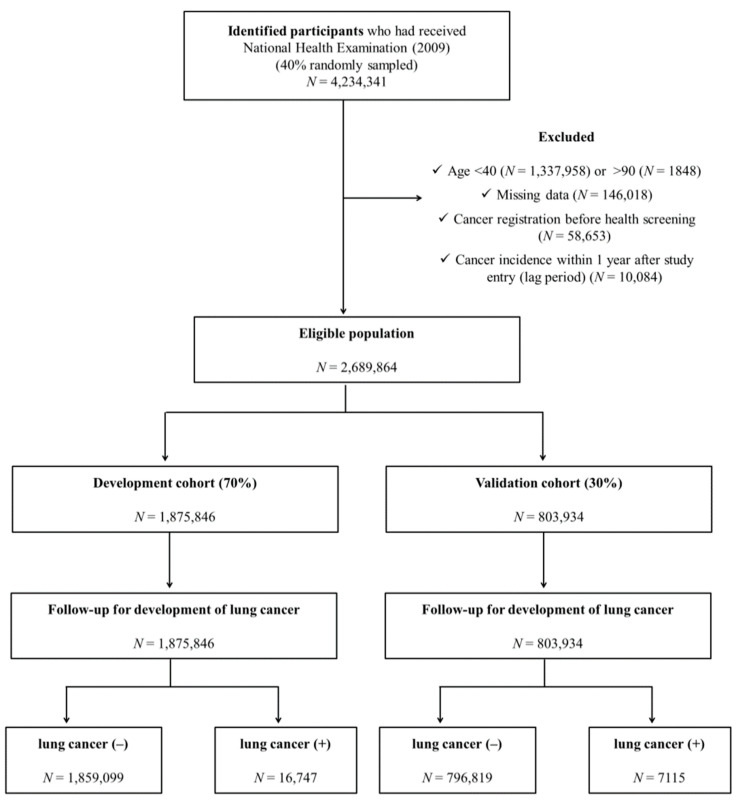
Study design summary.

Among all KNHI beneficiaries, the population for this study consisted of 40% randomly sampled participants who completed health examinations from 1 January 2009 to 31 December 2009. Among approximately 4 million subjects who participated in health screening in 2009, individuals <40 years of age (*n* = 1,337,958) or >90 years of age (*n* = 1848) or who had any type of cancer (confirmed C-code) before health screening (*n* = 58,653) were excluded in the present study. In addition, subjects diagnosed with any type of cancer within 1 year of study enrollment (*n* = 10,084) were excluded. Finally, a total of 2,689,864 subjects were eligible for participation in this study (Figure 1).

The development and validation datasets were formed by splitting the original cohort DB into two datasets. Approximately 70% of the eligible subjects were selected for the development cohort. For the internal validation cohort, the remaining 30% of the eligible subjects were extracted from the same DB using simple random sampling methods.

### 2.3. Predictor Variables

Among the available values in the KNHI DB, demographic information and personal clinical information were obtained, including age, sex, BMI, and socioeconomic status based on Medicaid insurance coverage. Age was divided into 5 groups (40–49, 50–59, 60–69, 70–79, and ≥80 years). Smoking intensity was categorized as follows: nonsmokers, <10, 10–20, 20–30, and ≥30 pack-years. Alcohol consumption was categorized as follows: nondrinkers, light (<15 g/day), moderate (15–30 g/day), and heavy drinkers (>30 g/day). The subjects were also classified into 5 groups based on the BMI category of WHO recommendations for Asians (<18.5, 18.5–22.9, 23.0–24.9, 25.0–29.9, and ≥30 kg/m^2^).

The presence of comorbidities was defined based on diagnostic codes with or without prescription of relevant medications or health checkup results: diabetes was defined as ICD-10 codes E11–E14 with at least one prescription of an antidiabetic medication or a fasting glucose level ≥126 mg/dL. Chronic obstructive pulmonary disease (COPD) was defined based on ICD-10 codes J43 (emphysema) and J44 (other COPD) within 1 year of enrollment. The presence of pulmonary tuberculosis (TB) (ICD10 codes A15–A19) within 1 year of enrollment was also included. Insurance coverage was assessed using monthly insurance premiums because insurance contribution is determined based on income level in Korea.

### 2.4. Lung Cancer as an Outcome

The incidence of lung cancer was defined based on diagnosis codes for lung cancer (C34) registered after baseline screening with inclusion in a special copayment reduction program for critical illness. In Korea, nearly all people apply for this program if they are diagnosed with cancer because a 5% copayment applies for the work-up and cancer treatment (vs. 20–30% for other common diseases). If the patients were clinically compatible with radiological findings of lung cancer, histopathological confirmation was obtained for qualification of the copayment reduction program, unless there was clinically evident advanced cancer for which no treatment was indicated. Therefore, cancer incidence in Korea is rarely omitted from this claims DB and is sufficiently reliable. To evaluate the occurrence of lung cancer among the included participants, the claims DB was monitored until 31 December 2018.

### 2.5. Development of Risk Prediction Model

Among potential risk factors for lung cancer, 9 variables that had good predictive abilities based on literature review were selected. Candidate predictors included age, sex, cigarette smoking (intensity), BMI (kg/m^2^) [23,24], alcohol consumption (intensity) [25], presence of diabetes mellitus (DM) [26], COPD (emphysema and chronic bronchitis) [27], pulmonary TB [28], and health insurance types (covered or not by Medicaid) [29]. Both crude and adjusted risks were explored for possible risk variables, and each variable was input into the model as a categorical variable. A multivariable model using the Cox proportional hazards model was developed using the times to event between the date of health examination and the date of first lung cancer diagnosis or follow-up termination, whichever came first. The proportional hazards assumption was evaluated by investigating Schoenfeld residuals with the logarithm of the cumulative hazard function based on Kaplan–Meier curves. Finally, the best-fit risk prediction model was built using backward selection.

**Figure 2:**
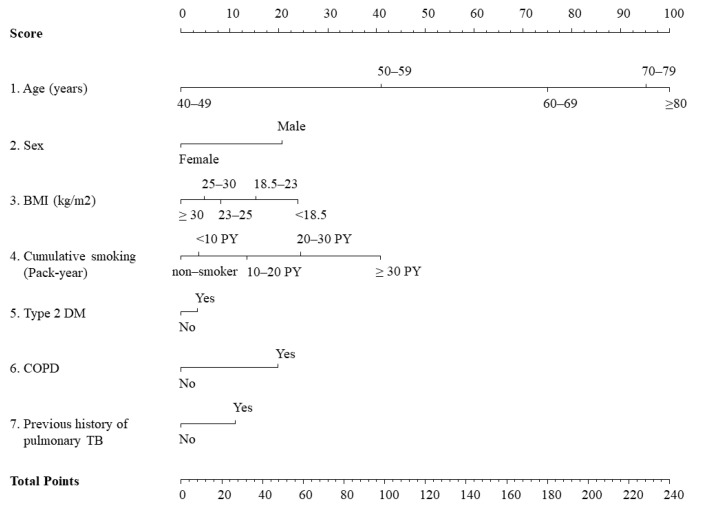
Nomogram for the 7-variable prediction model of lung cancer probability. BMI, body mass index; DM, diabetes mellitus; COPD, chronic obstructive pulmonary disease; TB, tuberculosis.

The 7 adopted predictors (age category, sex, BMI category, cumulative smoking intensity, presence of COPD, type 2 DM, and pulmonary TB) were applied as weighted risk scores based on the b coefficients for each risk factor in the final Cox proportional hazards by assigning scores ranging from 0 to 100 [30]. The total score, which was the sum of the scores for each of the 7 variables, ranged from 0 to 240. The detailed nomogram for lung cancer risk in our prediction model is presented in Figure 2.

### 2.6. Validation of the Risk Prediction Model

Performance of the model was evaluated with respect to discrimination and calibration.

Model discrimination was assessed using the concordance statistic (c-statistic) for survival data. ROC curves are concordance measures with c-statistic interpreting the probability of how closely the model predicts the risk of lung cancer for subjects who actually developed lung cancer compared with those who did not during follow-up. The prediction model is considered good when the discrimination is 0.60–0.80, and a value >0.80 is considered excellent [31]. Internal validation of model discrimination was assessed by calculating the bootstrap optimism-corrected c-statistic with 100 bootstrap replications [32].

Model calibration was assessed by plotting the mean predicted probability against the mean observed probability of lung cancer. Calibration ability refers to how closely the predicted probabilities agree numerically with the actual outcomes. The χ2 statistic was calculated by first dividing the data into deciles based on the predicted probabilities produced by the model in ascending order. Then, in each decile, the average predicted probabilities were compared with the actual lung cancer risk estimated using the Kaplan–Meier approach. The performance of the developed model was also tested on the validation dataset with regard to both discrimination and calibration.

### 2.7. Statistical Analyses

Descriptive data are presented as means ± standard deviation (SD) and frequencies as percentage (%). To evaluate the difference between the proportions or means of two variables, chi-square tests and Student’s *t*-tests were used. Incidence rates of lung cancer were estimated as events per 1000 person-years (PYs). A two-sided *p*-value <0.05 was considered statistically significant, and all analyses were performed using complete data only. All analyses were performed using SAS (version 9.4; SAS Institute, Cary, NC, USA).

## 3. Results

### 3.1. Clinical Characteristics of the Study Population in the Development and Validation Cohorts

Among 1,975,846 subjects in the development cohort, 16,747 individuals (0.85%) developed lung cancer during the follow-up period (mean, 8.2 years). The incidence rate of lung cancer was 1.09 per 1000 PYs. Compared with the subjects who did not develop lung cancer, the subjects who developed lung cancer were older and male. Greater smoking intensity and alcohol consumption were observed in patients who developed lung cancer (Table 1). Among the other 30% of the study population in the validation cohort (*n* = 803,934), the mean age was 54.2 years, and approximately 50% of the subjects were female. Among them, 7115 patients (0.89%) developed lung cancer during the follow-up period (mean, 8.2 years). The clinical characteristics of the validation cohort were similar to those of the development cohort, including age, sex, and BMI, as well as lung cancer incidence rate (1.08/1000 PYs) (Table 1).

### 3.2. Selection of Predictor Variables for the Prediction Model

The crude and adjusted hazard ratios (aHRs) for nine variables in the model are presented in Table 2. The HR was higher based on age group and persisted after adjusting for all listed variables (model 1): sex, exercise level, BMI, smoking and drinking habits, presence of diabetes, COPD, previous history of pulmonary TB, alcohol consumption, and insurance coverage. Female sex (aHR, 0.56; 95% CI: 0.53–0.58) was also a significant predictive factor for the development of lung cancer. After categorization into five groups, smoking intensity was significantly associated with lung cancer risk with a linear trend (for <10 pack-years, aHR, 1.12; 95% CI: 1.05–1.21; and for ≥30 pack-years, aHR, 3.07; 95% CI: 2.93–3.22) (model 1). After BMI categorization into five groups, an inverse relationship with a linear trend was observed compared with normal BMI (18.5–22.9 kg/m^2^) in Asians (<18.5 kg/m^2^; aHR, 1.26; 95% CI: 1.16–1.36; and ≥30 kg/m^2^; aHR, 0.66; 95% CI: 0.59–0.73) (model 1). The presence of COPD (aHR, 1.70; 95% CI: 1.62–1.79) or previous history of pulmonary TB (aHR, 1.34; 95% CI: 1.22–1.47) also showed increased risk for lung cancer incidence. However, alcohol consumption (aHR for heavy drinkers, 0.97; 95% CI: 0.91–1.02) and coverage by Medicaid (aHR, 1.05; 95% CI: 0.97–1.13) were not significant factors in model 1. To determine the best-fit model using backward elimination methods, alcohol consumption and coverage by Medicaid were finally eliminated from the final model (model 2).

### 3.3. Development of Scores for Lung Cancer Prediction

**Figure 3:**
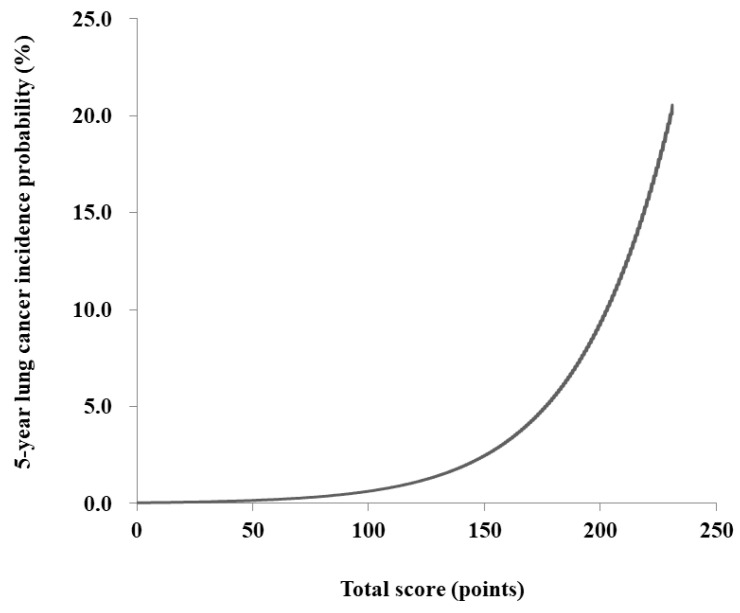
The 5-year incidence probability of lung cancer based on the total score.

The risk prediction model for lung cancer was translated into a risk score nomogram (Figure 2). The sum of the scores for seven variables ranged from 0 to 240. Individual risk can be estimated as follows: for example, a male (21 points), 60 years of age (75 points), currently smoking >20 pack-years (24 points), without chronic lung disease (0 point), or past history of pulmonary TB (0 points) but with type 2 DM (3 points), would have 123 points (Figure 2, Table S1). The 5-year lung cancer incidence probability for this male is estimated to be 1.2%. If the total score is >200 points, lung cancer incidence probability increases up to >10.0% (Figure 3).

**Figure 4:**
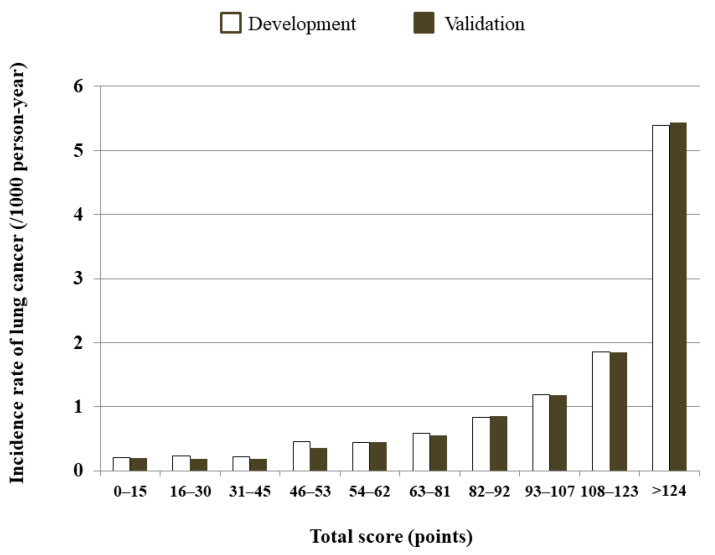
Predicted 5-year lung cancer incidence rate (per 1000 person-years (PYs)) based on decile score using the development and validation cohorts.

The decile score showed that the subjects in the highest decile (total score >124) had the highest incidence rate of 5.39 per 1000 PYs (Figure 4, Table S2).

### 3.4. Validation of the Risk Model

Our risk prediction model showed good discrimination (c-statistic, 0.810; 95% CI: 0.801–0.819). When the performance of the developed model was tested on the validation cohort, the c-statistic for 5-year prediction of lung cancer incidence was 0.825 (95% CI: 0.810–0.840). 

The relationship between model-predicted and actual lung cancer development correlated well in the calibration plot (Figure S1). Compared with the dashed line representing the performance of an ideal nomogram, the solid line representing the actual outcome showed a nearly 45-degree line, indicating that this model corresponded well with an absolute lung cancer event.

## 4. Discussion

A risk prediction model for lung cancer in Korea was developed and validated using the KNHI DB. The performance of the model was good with competent discrimination with a c-statistic of 0.810 (95% CI: 0.801–0.819) and calibration ability. To establish clinically relevant and meaningful models for the general population, the use of easily accessible and modifiable risk factors for lung cancer has been emphasized. Each of the seven variables used in the 5-year lung cancer risk model consisted of clinically important but easily applicable variables. We showed that this prediction model provides accurate risk prediction for lung cancer in a population-based cohort and is applicable to health examinees or the general adult population.

Since a Korean national lung cancer screening program has been in operation since 2019, there are several efforts to identify the appropriate population recommended for screening and to maximize the efficacy of the screening program. Of them, the Korean Cancer Society and the Korean Foundation for Cancer Research has driven a study project to provide aid for self-decisions on participating in lung cancer screening, and our lung cancer risk model for Koreans has been developed. Healthcare providers can advise early screening for lung cancer or lifestyle modification, including smoking cessation, based on the estimated risk using this prediction model. We believe that interactive approaches between healthcare providers and examinees using an easily accessible and visualized risk score can be used for the development of health policies for lung cancer prevention.

The crude lung cancer incidence rates in study participants were compared with those in subjects in the general population in Korea within identical age ranges. Study participants having follow-ups between their health examinations in 2009 and December 2018 were compared with those included in the 2017 registry of cancer incidence in the Korean population [33] to determine the age-specific rates per 100,000 PYs in each age group. The results for the study participants and general population were as follows: 40–49 years of age (24.6 vs. 22.3), 50–59 years of age (80.0 vs. 91.8), 60–69 years of age (206.7 vs. 291.3), 70–79 years of age (345.2 vs. 575.6), and >80 years of age (378.1 vs. 651.0). Because the number of subjects >90 years of age included in the registry and the number of subjects >80 years of age who participated in health examinations in our study cohort were relatively low, this model was representative of the Korean population, although the incidence rates of the study participants were slightly lower.

### 4.1. Previous Lung Cancer Prediction Models

Previous prediction models from Western countries have estimated individual lung cancer risk with good predictive abilities [8,9,10,11,12,13,14]. Although relatively few prediction models have been developed in Asian countries, a prospective cohort study of 395,875 subjects in Taiwan consistently predicted individual lung cancer risk with a c-statistic of 0.73–0.85 regardless of smoking status after integrating the risk factors of family history, tumor markers (carcinoembryonic antigen (CEA) or alpha fetoprotein (AFP)), and lung function (FEV1) [34]. A Korean prediction model [17] with modifiable risk factors also showed accuracy with a c-statistic of 0.87 in predicting 8-year lung cancer risk in men. A previous Korean model included family history of lung cancer, but it was not significant in the final model. Instead of fasting glucose level, DM diagnosis evaluated in our model showed a similar risk level for lung cancer and appeared more intuitive for obtaining an immediate assessment in a clinical setting or self-assessment. Regarding discrimination ability, our model is comparable to or better than previous models without integrating other genetic or laboratory findings.

Smoking exposure is the most important established risk factor for lung cancer incidence [35,36,37,38]. In previous models, smoking-related variables were used to estimate smoking exposure: smoking intensity (UK Biobank [14], PLCO [8], EPIC [13], Spitz [12], Bach [15], Pittsburgh [16], and Korean [17] models), duration (Spitz [12], LLP [11], PLCO [8], Bach [15], and Pittsburgh models [16]), age when started smoking (Spitz [12], EPIC [13], and Korean [17] models), age when stopped smoking (Spitz model [12]), and/or time since smoking cessation (Bach [15] and UK Biobank [14] models). In several studies, individual smoking exposure was titrated based on spline effects of pack-years, smoking duration, and smoking quit-time duration [8,17]. Similar to previous models, smoking intensity estimated based on pack-years, a well-known reliable parameter for smoking exposure, adequately predicted lung cancer incidence in Koreans with a clear dose-response manner in the present study. Furthermore, regarding decline in discrimination by including noncurrent smokers in the model [8,11,12], our model with noncurrent smokers and the calculated risk relative to them could be expanded to the general adult population with an excellent performance. Immediate calculations based on self-assessment could help subjects readily use lung cancer prediction models.

### 4.2. Predictor Variables in Lung Cancer

In addition to age and smoking exposure, candidate variables in epidemiologic studies for lung cancer incidence included sex, with higher risk in men; BMI, with an inverse association [23]; underlying pulmonary diseases such as COPD; pulmonary infectious diseases, including bronchitis [11,27], TB [28], and pneumonia [39]; and individual lung function represented by FEV1 [14]. Other variables included nicotine addiction [36], occupational exposure and secondhand smoking [12], inflammatory markers [34], allergic conditions [40,41], and type 2 DM [26], as well as family history of lung cancer [42,43,44]. Because we wanted to develop a simple and easily accessible prediction model for adults, parameters that needed further questionnaires or laboratory tests to complete risk calculation were not considered. Future prediction models including environmental exposure or genetic factors should be developed.

In the present study, fewer women were current smokers than men, and risk of lung cancer was lower in women (aHR, 0.60; 95% CI: 0.52–0.71) than in men. After selection of predictor variables, male sex was a significant predictor for lung cancer incidence, which was consistent with previous models. Regarding the higher risk in men, previous models have also included sex in the model as a predictor variable (Bach [15], LLP [11], UK Biobank [14], and EPIC [13] models) based on stratification (PLCO model [8]) or restriction to men (Spitz [12] and Korean [17] models). Regarding the status of smoking rates in women compared with the decline in men over decades [45,46], the risk difference based on sex should be a factor of interest in lung cancer prediction in Korea.

In addition, lower BMI was mainly associated with lung cancer risk among the statistically selected seven variables. Lean body weight represented by lower BMI indicated increased risk of lung cancer. In previous epidemiological studies, higher BMI was shown to be associated with lower overall lung cancer risk, which was further confirmed in meta-analyses [23,24]. To clarify the confounding effects of smoking, a meta-analysis of nonsmokers was performed to investigate the association between BMI and lung cancer risk. Zhu et al. reported that an inverse linear dose-response relationship was observed between BMI and lung cancer risk in never smokers [47]. In the present study, subjects with lower BMI (<18 kg/m^2^) had the highest score (score 24) even after adjusting for smoking intensity in a statistically fitted model. The inverse relationship between BMI and lung cancer was consistently present in a previous Korean prediction model [17]. The possible plausibility of this inverse association between BMI and lung cancer risk can be explained by the effect of adipose tissue on DNA adducts that are associated with storage and metabolism of carcinogen [48,49]. In addition, the linkage of excess body fat to increase in insulin level might inhibit carcinogenesis by suppressing apoptosis and improving immune function [50,51].

Several studies have been performed regarding comorbidities associated with lung cancer incidence. Regarding the presence of COPD, in a large pooled case–control study, chronic bronchitis and emphysema increased lung cancer risk by 30% after accounting for smoking [27]. A potential explanation for the increase in lung cancer risk is the inflammatory response to chronic bronchitis and emphysema, which is conducive to tumor initiation [52]. Increases in genetic mutations, angiogenesis [53], and antiapoptotic signaling [54] are potential processes through which inflammation may increase the risk of cancer development. However, a prediction model in a prospective cohort study of UK Biobank previously showed that lung function was incorporated with lung cancer incidence [14]. Pulmonary TB has also been postulated to have a causal association with lung cancer. In a previous study including 1 million people with a 16-year median follow-up in Korea, which has a high prevalence of TB, the presence of underlying TB was significantly associated with increased risk of lung cancer [28]. Due to a modest increase in the lung cancer risk without an effect modification by smoking, the authors suggested that underlying TB can be incorporated into a lung cancer model, especially in Korea, where a high prevalence of TB exists. This association can be evidenced by chronic inflammation, oxidative stress, or fibrosis [55,56]. The presence of pulmonary TB in this model showed increased lung cancer risk, although the score strength was relatively small.

In our model, type 2 DM was also associated with a small risk of lung cancer incidence. Subjects who were comorbid with type 2 DM showed a 20% increased risk of lung cancer with an incidence rate of 1.70 (per 1000 PYs). In a meta-analysis, type 2 DM was significantly associated with increased risk of lung cancer compared with nondiabetic controls after adjusting for smoking (RR, 1.11; 95% CI: 1.02–1.20) [26]. Hyperinsulinemia, insulin resistance, and chronic inflammation may contribute to lung structural damage and be associated with the neoplastic process [57]. Although the presence of listed comorbidities showed that the increased lung cancer risk was relatively small, there are important health implications for motivating patients with comorbidities to participate in lifestyle modification based on individualized lung cancer risk.

### 4.3. Limitations

The present study has several limitations. First, smoking habits included in the model were based only on intensity and not on duration or age at smoking initiation. However, pack-year estimation is a representative measure for smoking exposure, and pack-years in our model showed good performance. Second, underestimation of smoking in women may have occurred because female smoking has a negative connotation in the Korean culture. Third, severity status of disease comorbidities was not input into the model. Fourth, information on histopathologic type or stage of lung cancer was not available in our model.

## 5. Conclusions

We developed a multivariable risk model to predict lung cancer incidence in Korean adults. The scores in this prediction model may serve as a tool to screen high-risk individuals who would benefit from participating in lung cancer screening in a clinical setting. Physicians or healthcare providers can motivate participants with or without comorbidities to reduce their risk by quitting smoking or maintaining proper body weight not only for overall health improvement but also for prevention of future lung cancer based on the risk calculated using this model. Future studies identifying whether this model helps subjects in making decisions to participate in lung cancer screening or initiating lifestyle modifications based on their individualized risk should be performed.

## Figures and Tables

**Table 1:** Baseline characteristics of cohort population based on lung cancer incidence in the development and validation cohorts.

	Developmental Cohort (*n* = 1,975,846)			Validation Cohort (*n* = 803,934)		
	Lung Cancer Did Not Develop (*n* = 1,859,099)	Lung Cancer Developed (*n* = 16,747)	*p*-Value ^a^	Lung Cancer Did Not Develop (*n* = 796,819)	Lung Cancer Developed (*n* = 7115)	*p*-Value ^a^
Age (years) (*N*, %)						
40–49	725,609 (39.0)	1481 (8.8)	<0.001	310,720 (39.0)	561 (7.9)	<0.0001
50–59	578,958 (31.1)	3859 (23.0)		247,804 (31.1)	1674 (23.5)	
60–69	361,299 (19.4)	6193 (37.0)		155,121 (19.5)	2659 (37.4)	
70–79	168,698 (9.1)	4606 (27.5)		72,753 (9.1)	1989 (28.0)	
≥80	24,535 (1.3)	608 (3.6)		10,421 (1.3)	232 (3.3)	
Sex (male) (*N*, %)	926,036 (49.8)	11,989 (71.6)	<0.001	396,541 (49.8)	5200 (73.1)	<0.0001
BMI (kg/m^2^) (*N*, %)						
<18.5	40,779 (2.2)	670 (4.0)	<0.001	17,599 (2.2)	260 (3.65)	<0.0001
18.5–23	668,716 (36.0)	6668 (39.8)		286,694 (36.0)	2839 (39.9)	
23–25	496,918 (26.7)	4329 (25.9)		212,892 (26.7)	1868 (26.3)	
25–30	590,430 (31.8)	4714 (28.2)		252,927 (31.7)	2004 (28.2)	
≥30	62,256 (3.4)	366 (2.2)		26,707 (3.4)	144 (2.0)	
Smoking (pack-years) (*N*, %)						
Nonsmoker	1,193,868 (64.2)	7247 (43.3)	<0.001	512,328 (64.3)	2973 (41.8)	<0.0001
<10	158,726 (8.5)	979 (5.9)		68,088 (8.5)	423 (6.0)	
10–20	185,327 (10.0)	1571 (9.4)		79,402 (10.0)	646 (9.1)	
20–30	157,04 7(8.5)	1899 (11.3)		67,004 (8.4)	861 (12.1)	
≥30	164,131 (8.8)	5051 (30.2)		69,997 (8.8)	2212 (31.0)	
Alcohol drinking (*N*, %)						
Nondrinker	1,085,573 (58.4)	9421 (56.3)	<0.001	465,956 (58.5)	3940 (55.4)	<0.0001
Light drinker	453,362 (24.4)	3653 (21.8)		194,041 (24.3)	1568 (22.0)	
Moderate drinker	183,947 (9.9)	1903 (11.3)		78,682 (9.9)	828 (11.6)	
Heavy	136,217 (7.3)	1770 (10.6)		58,140 (7.3)	779 (11.0)	
Type 2 DM (yes) (*N*, %)	220,692 (11.9)	3028 (18.1)	<0.001	95,092 (11.9)	1329 (18.7)	<0.0001
COPD (yes) (*N*, %)	64,554 (3.5)	1997 (11.9)		28,004 (3.5)	847 (11.9)	
Pulmonary TB (yes) (*N*, %)	22,083 (1.2)	485 (2.9)		9688 (1.2)	228 (3.2)	
Insurance coverage (Medicaid) (*N*, %)	76,054 (4.1)	738 (4.4)	0.04	32,830 (4.1)	311 (4.4)	0.29

Abbreviations: BMI, body mass index; DM, diabetes mellitus; COPD, chronic obstructive pulmonary disease; TB, tuberculosis. ^a^ Tested using chi-square test for categorical variables.

**Table 2:** Hazard ratios (HRs) and 95% confidence interval (CI) for lung cancer incidence.

	Number of Subjects	Event	Follow-Up (PYs)	IR	Crude HR (95% CI)	Model 1 aHR (95% CI)	Model 2 aHR (95% CI)
Age (years)							
40–49	727,090	1481	6027, 618.8	0.25	1 (ref)	1 (ref)	1 (ref)
50–59	582,817	3859	4822, 231.1	0.80	3.26 (3.07–3.46)	3.03 (2.86–3.22)	3.05 (2.87–3.24)
60–69	367,492	6193	2996, 657.1	2.07	8.42 (7.95–8.91)	7.61 (7.18–8.07)	7.71 (7.27–8.17)
70–79	173,304	4606	1334, 442.8	3.45	14.15 (13.34–15.00)	13.11 (12.33–13.93)	13.35 (12.57–14.19)
80–89	25,143	608	160, 846.8	3.78	15.87 (14.44–17.44)	14.88 (13.51–16.39)	15.21 (13.82–16.75)
Sex							
Male	938,025	11,989	7597, 638.5	1.58	1 (ref)	1 (ref)	1 (ref)
Female	937,821	4758	7744, 157.9	0.61	0.39 (0.38–0.40)	0.56 (0.53–0.58)	0.60 (0.52–0.71)
BMI (kg/m^2^)							
<18.5	41,449	670	321, 011.2	2.09	1.73 (1.60–1.88)	1.26 (1.16–1.36)	1.20 (0.90–1.59)
18.5–23	675,384	6668	5504, 084.6	1.21	1 (ref)	1 (ref)	1 (ref)
23–25	501,247	4329	4113, 813.5	1.05	0.87 (0.84–0.90)	0.82 (0.79–0.86)	0.86 (0.75–0.98)
25–30	595,144	4714	4889, 253.9	0.96	0.80 (0.77–0.83)	0.75 (0.72–0.78)	0.76 (0.67–0.86)
≥30	62,622	366	513, 633.42	0.71	0.59 (0.53–0.65)	0.66 (0.59–0.73)	0.65 (0.45–0.94)
Smoking (pack-year)							
Nonsmoker	1,201,115	7247	9874, 552.2	0.73	1 (ref)	1 (ref)	1 (ref)
<10	159,705	979	1307, 649.6	0.75	1.023 (0.96–1.09)	1.12 (1.05–1.21)	1.28 (1.01–1.63)
10–20	186,898	1571	1524, 583.2	1.03	1.41 (1.33–1.49)	1.47 (1.38–1.56)	1.51 (1.22–1.88)
20–30	158,946	1899	129, 0218.4	1.47	2.01 (1.91–2.12)	1.98 (1.87–2.10)	2.53 (2.09–3.06)
≥30	169,182	5051	1344, 793.1	3.76	5.14 (4.96–5.33)	3.07 (2.93–3.22)	3.47 (2.96–4.07)
Alcohol consumption							
Nondrinker	1,094,994	9421	8951, 014.5	1.05	1 (ref.)	1 (ref.)	-
Light	457,015	3653	3753, 142.2	0.97	0.93 (0.89–0.96)	0.89 (0.85–0.92)	
Moderate	185,850	1903	1518, 858.3	1.05	1.19 (1.14–1.25)	0.94 (0.89–0.99)	
Heavy	137,987	1770	1118, 511.4	1.58	1.51 (1.43–1.59)	0.96 (0.91–1.02)	
Presence of type 2 DM							
No	1,652,126	13,719	13,563, 423.2	1.01	1 (ref)	1 (ref)	1 (ref)
Yes	223,720	3028	1,778,373.3	1.70	1.67 (1.62–1.76)	1.09 (1.05–1.14)	1.22 (1.07–1.40)
Presence of COPD							
No	1,809,295	14,750	14,830, 410.7	0.99	1 (ref)	1 (ref)	1 (ref)
Yes	66,551	1997	511, 385.8	3.91	3.94 (3.76–4.13)	1.70 (1.62–1.79)	1.70 (1.44–2.00)
Presence of pulmonary TB							
No	1,853,278	16,262	15,165, 257.7	1.07	1 (ref)	1 (ref)	1 (ref)
Yes	22,568	485	176, 538.8	2.75	2.57 (2.35–2.81)	1.34 (1.22–1.47)	1.62 (1.23–2.14)
Insurance coverage							
Non-Medicaid	1,799,054	16,009	14,712, 765.1	1.09	1 (ref)	1 (ref)	-
Medicaid	76,792	738	629, 031.4	1.17	1.08 (1.00–1.16)	1.05 (0.97–1.13)	

Abbreviations: PYs, person-years; HR, hazard ratio; IR, incidence rate per 1000 person-years; aHR, adjusted hazard ratio; CI, confidence interval; BMI, body mass index; DM, diabetes mellitus; COPD, chronic obstructive pulmonary disease; TB, tuberculosis. Model 1: adjusted for all possible predictor variables listed in the table. Model 2: adjusted for selected predictor variables using backward selection.

## Data Availability

The datasets used for the current study are available from the corresponding author on reasonable request.

## Supplementary Materials

The following are available online at https://www.mdpi.com/article/10.3390/cancers13143496/s1, Table S1: Scores for each risk factor category, Table S2: Predicted incidence rate (per 1,000 person-years) based on the development and validation cohorts, Figure S1: Calibration plots between predicted and observed 5-year lung cancer development.
