# Supplementary material for: Individual 5-Year Lung Cancer Risk Prediction Model in Korea Using a Nationwide Representative Database
Source: Cancers (Basel). 2021 Jul 13;13(14):3496. doi: 10.3390/cancers13143496 (PMC8307783; doi:10.3390/cancers13143496)
Supplement: Supplementary file 1 [file cancers-13-03496-s001.zip › cancers-1237994-supplementary.pdf]

cancers

MDPI

Supplementary Materials

Individual 5-Year Lung Cancer Risk Prediction Model in Korea Using a Nationwide Representative Database

Yohwan Yeo, Dong Wook Shin, Kyungdo Han, Sang Hyun Park, Keun-Hye Jeon, Jungkwon Lee, Junghyun Kim and Aesun Shin

Table S1. Scores for each risk factor category.

No.	Variables	Categories and scores				
1.	Age (years)	40–49	50–59	60–69	70–79	≥ 80
		0	41	75	95	100
2.	Sex	Female	Male			
		0	21			
3.	Smoking (pack-years)	Non-smoker	< 10	10–20	20–30	≥ 30
		0	4	13	24	41
4.	BMI (kg/m2)	< 18.5	18.5–23	23–25	25–30	≥ 30
		24	15	8	5	0
5.	Type 2 DM	No	Yes			
		0	3			
6.	COPD	No	Yes			
		0	20			
7.	Previous history of pulmonary TB	No	Yes			
		0	11			

BMI, body mass index; DM, diabetes mellitus; COPD, chronic obstructive pulmonary disease; TB, tuberculosis.

Table S2. Predicted incidence rate (per 1,000 person-years) based on the development and validation cohorts.

Decile		1st	2nd	3rd	4th	5th	6th	7th	8th	9th	10th
Points		0–15	16–30	31–45	46–53	54–62	63–81	82–92	93–107	108–123	124–
Incidence rate of lung cancer (per 1,000 person-years)	Development cohort	0.20	0.23	0.22	0.45	0.45	0.58	0.83	1.19	1.85	5.39
	Validation cohort	0.19	0.19	0.19	0.36	0.45	0.56	0.85	1.18	1.84	5.43

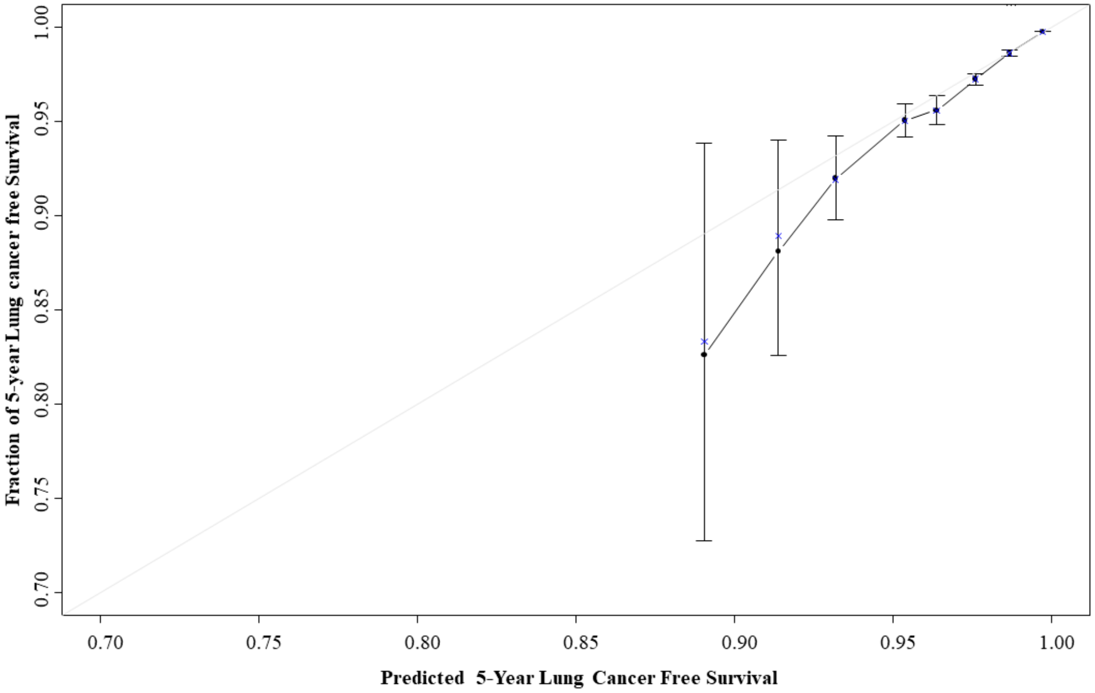

Figure S1. Calibration plots between predicted and observed 5-year lung cancer development.
